# Characterization of a functional insertion sequence IS*Sau2* from *Staphylococcus aureus*

**DOI:** 10.1186/s13100-018-0108-5

**Published:** 2018-01-16

**Authors:** Liangliang Wang, Wei Si, Huping Xue, Xin Zhao

**Affiliations:** 1 College of Animal Science and Technology, Northwest A&F University, No.3 Taicheng Road, Yangling, 712100 Shaanxi Province People’s Republic of China; 2 School of Pharmaceutical Sciences, Tsinghua University, Beijing, People’s Republic of China; 3 Tsinghua University-Peking University Joint Center for Life Sciences, Beijing, People’s Republic of China; 4 Department of Animal Science, McGill University, Quebec, Canada

**Keywords:** IS*Sau2*, Functional insertion sequence, IS*150*, Transposition frequency

## Abstract

**Background:**

IS*Sau2* has been suggested as a member of the IS*150* f subgroup in the IS3 family. It encodes a fusion transposase OrfAB produced by programmed − 1 translational frameshifting with two overlapping reading frames *orfA* and *orfB*. To better characterize IS*Sau2*, the binding and cleaving activities of the IS*Sau2* transposase and its transposition frequency were studied.

**Results:**

The purified IS*Sau2* transposase OrfAB was a functional protein in vitro since it bound specifically to IS*Sau2* terminal inverted repeat sequences (IRs) and cleaved the transposon ends at the artificial mini-transposon pUC19-IRL-gfp-IRR. In addition, the transposition frequency of IS*Sau2* in vivo was approximately 1.76 ± 0.13 × 10^− 3^, based on a GFP hop-on assay. Furthermore, OrfB cleaved IRs with the similar catalytic activity of OrfAB, while OrfA had no catalytic activity. Finally, either OrfA or OrfB significantly reduced the transposition of IS*Sau2* induced by OrfAB.

**Conclusion:**

We have confirmed that IS*Sau2* is a member of IS*150*/IS*3* family. The IS*Sau2* transposase OrfAB could bind to and cleave the specific fragments containing the terminal inverted repeat sequences and induce the transposition, suggesting that IS*Sau2* is at least partially functional. Meanwhile, both OrfA and OrfB inhibited the transposition by IS*Sau2*. Our results will help understand biological roles of IS*Sau2* in its host *S. aureus*.

## Background

Insertion sequences are ubiquitous in prokaryote and eukaryotes. They exert a major effect on genome evolution. Previously, we have suggested that an insertion sequence IS*Sau2* in *Staphylococcus aureus* was probably a member of the IS*150* subgroup in the IS*3* family based on the sequence structure and searching results from the ISfinder database [1]. Members of the IS*3* family have a general structure, consisting of a single transposase gene flanked by terminal inverted repeats (IRs) and the transposase gene contains two open reading frames, *orfA* and *orfB*. The transposase contains a DNA-binding helix-turn-helix (HTH) motif which specifically recognizes the transposon inverted repeats [2] and a DDE domain which catalyzes transposition reactions [3]. The IS*3* family can be further divided into six subgroups (IS*150*, IS*407*, IS*51*, IS*3*, IS*2* and IS*911*) based on the structure of insertion sequences and alignment of their OrfB sequences [4]. Whether IS*Sau2* functions as a member of the IS*150* subgroup remains to be determined.

While searching the ISfinder database, 11 subgroups/groups of insertion sequences contain two open reading frames, *orfA* and *orfB.* Among them, 8 (IS*1*, IS*150*, IS*407*, IS*51*, IS*3*, IS*2*, IS*427*, and IS*630*) definitely use OrfAB as the transposase. At the same time, which protein (OrfA, OrfB or OrfAB) functions as a transposase in the other 3 subgroups/groups (IS*21*, IS*605*, IS*607*) remains elusive [4, 5]. For the vast majority of insertion sequences in the IS*3* family, the OrfAB transposase is produced by programmed − 1 translational frameshifting with the motif A_n_G as the frameshifting region [5, 6]. Translational frameshifting is essential for expression of OrfAB in the IS*3* family [7]. A bioinformatics analysis revealed that IS*Sau2* contains both *orfA* and *orfB* [8]. A frameshift could occur at the A_6_G sequence site to produce a single functional OrfAB. This needs to be experimentally confirmed.

The transposition of IS*3*/IS*150* subgroup elements, catalyzed by the OrfAB transposase, occurs by a cut and paste mechanism. In the first step of transposition, the transposase OrfAB specifically binds to one IR and cleaves it to generate a “figure of eight” loop [9–11]. In the second step of transposition, the figure of eight is processed into a transposon circle and completes the transposition process to another position of the genome. However, only a few studies have proved the transposition function of IS*150* elements either in vitro or in vivo. To better understand the biological activity of IS*Sau2*, the binding and cleaving activities of the IS*Sau2* transposases in vitro and the transposition of IS*Sau2* in vivo deserve exploration.

Besides OrfAB, OrfA and OrfB are also produced during the transposition of the IS*3* family suggesting potential roles of OrfA and OrfB for transposition in nature. OrfA contains a HTH motif and is considered to compete with OrfAB for the DNA binding site, while OrfB might compete for the catalytic site to reduce the transposase activity [4]. It has been reported that both OrfA and OrfB of the IS*3* subgroup inhibited transposition, based on generation of circles or linear molecules using an artificial plasmid product in vitro [10]. In addition, OrfB of IS*629*, a member of the IS*3* family, was also able to reduce the transposition frequency [12]. However, there was no study on the function of OrfA or OrfB of the IS*150* subgroup. It would be interesting to detect whether the OrfA or OrfB of IS*Sau2* has the same inhibition activity.

In our previous work, the distribution and sequence diversity of IS*Sau2* were determined [1]. The goals of this study were to determine binding and cleaving activities of the transposase OrfAB of IS*Sau2* and its transposition frequency and to investigate the inhibitory function of OrfA and OrfB in the transposition of IS*Sau2*.

## Results

### IS*Sau2* belongs to the IS*150* subgroup of the IS*3* family

**Fig. 1:**
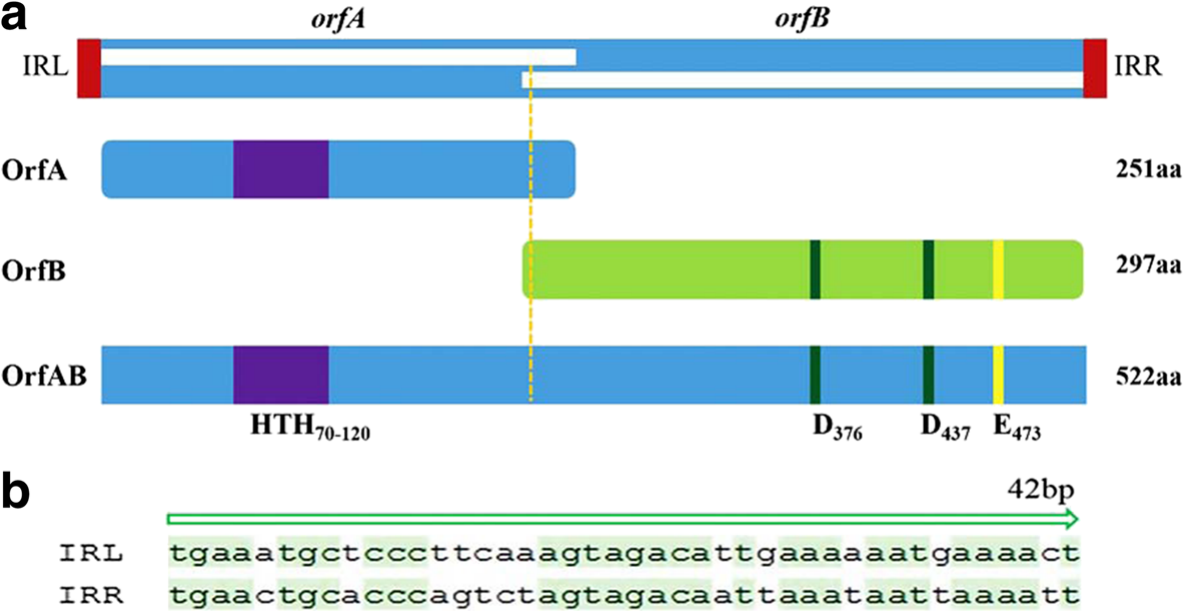
The structure of IS*Sau2*. **a** IS*Sau2* encodes a fusion protein (OrfAB, transposase), which contains two proteins OrfA (251aa) and OrfB (297aa), flanked by two imperfect inverted repeats (IRL and IRR). The frameshift site is shown as a dotted line. A HTH motif (70-120aa) is designated as a purple rectangle, and a DDE catalytic domain is also indicated. **b** The nucleotide sequences of assumed IRL and IRR are aligned

**Fig. 2:**
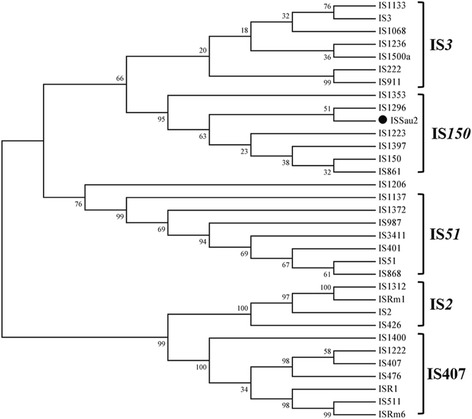
A dendrogram based on alignment of the amino acid sequences of predicted OrfB proteins from different IS*3* elements. The tree was constructed by MEGA 5.2 using the Neighbor-Joining method with default parameters. Bootstrap values were obtained from 1000 repetitions. The number at each node is the percentage of bootstrapped tree in which the insertion sequences to the right of the node were clustered. The major groups are indicated by brackets at the right. Obviously, the IS*Sau2* is nested within the IS*150* subgroup

IS*Sau2* from bovine *S. aureus* isolates was flanked by imperfect inverted 42 bp nucleotide repeats (IRL and IRR) (Fig. 1). Examination of IS*Sau2* OrfAB revealed a HTH motif within the N-terminal region and an Aspartate-Aspartate-Glutamate (DDE) catalytic domain in the C-terminal of OrfAB. There were 60 amino acid residues between the two D residues and 35 amino acid residues between the second D and E residues. A lysine (K) residue was six amino acids downstream of the E residue. For members of the IS*150* subgroup, lysine (K) residue should be six amino acids downstream of the E residue. To sub-categorize IS*Sau2*, a phylogenetic tree was constructed based on the amino acid sequence alignment of OrfB (Fig. 2). The result from the phylogenetic analysis confirmed that IS*Sau2* belongs to the IS*150* subgroup of the IS*3* family.

### Translational frameshift of OrfAB occurred in IS*Sau2*

**Fig. 3:**
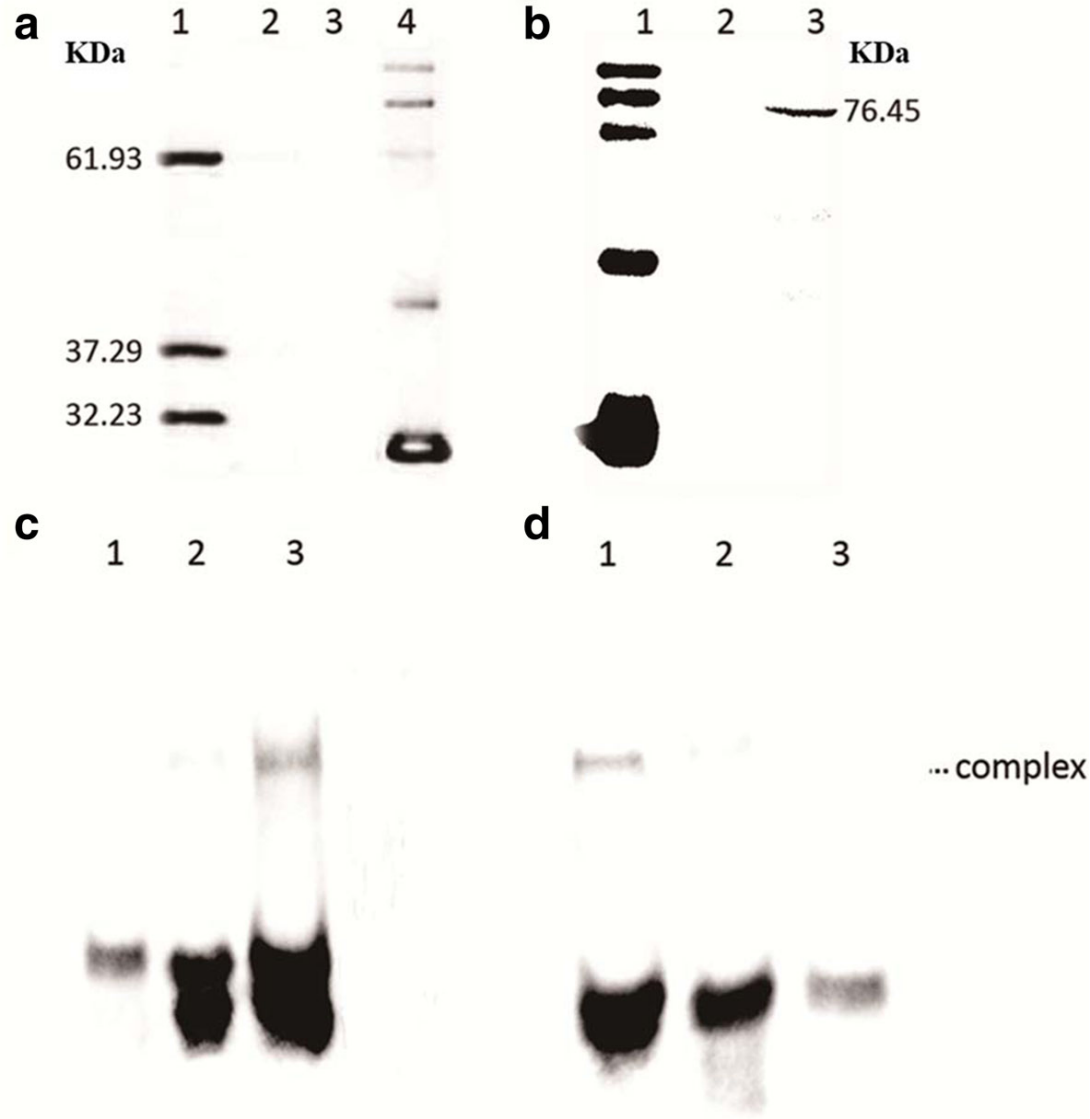
Western blot analysis of IS*Sau2* and EMSA analysis of binding of OrfAB to inverted repeats. **a** Western blot analysis of the protein extracts from BL21 with pET28-ISSau2. Lane 1, protein extracts from BL21 with pET28-ISSau2; lane 2, protein extracts from BL21 with pET28b as a negative control; lane 3, protein extracts from BL21, as a blank control; lane 4, Protein Marker. **b** Western blot analysis of the protein extracts from BL21 with pET32-OrfAB. Lane 1, Protein Marker; lane 2, protein extracts from BL21 with pET32a as a negative control; lane 3, protein extracts from BL21 with pET32-OrfAB. **c** EMSA analysis of binding of OrfAB to left inverted repeat (IRL). Lane 1, biotin-labeled IRL only; lane 2, biotin-labeled IRL incubated with protein extracts from BL21 with pET32a as a negative control; lane 3, biotin-labeled IRL incubated with purified protein OrfAB (0.42 μg). **d** EMSA analysis of binding of OrfAB to right inverted repeat (IRR). Lane 1, biotin-labeled IRR incubated with purified protein OrfAB (0.42 μg); lane 2, biotin-labeled IRR incubated with protein extracts from BL21 with pET32a as a negative control; lane 3, biotin-labeled IRR only

To verify the frameshifting phenomenon during expression of IS*Sau2*, the whole transposase sequence was inserted into pET28b to create ISSau2-pET28b, which was transformed into *E. coli* BL21 (DE3) for expression. As shown in Fig. 3a, three proteins were expressed with similar concentrations and expected to be OrfAB, OrfB and OrfA from top to bottom. The results indicated that OrfAB of IS*Sau2* was produced by − 1 translational frameshifting.

### OrfAB bound to IS*Sau2* IRs in vitro

In order to ascertain the binding between transposase OrfAB and the target DNA, an IS*Sau2* mutant, containing a single guanine insertion in the A_6_G sequence to generate A_6_G_2_, was inserted into pET32a to create pET32-OrfAB and expressed in *E. coli* BL21 (DE3). The transposase OrfAB was expressed according to the Western blot analysis (Fig. 3b) and purified.

To test whether the transposase can bind to DNA, an electrophoretic mobility shift assay (EMSA) was carried out with the purified transposase OrfAB and the biotin-labeled 42 bp oligonucleotides (IRL and IRR) which included the putative transposase binding sites. Transposase OrfAB bound to IRL42 in the presence of an excess of the poly (dI-dC) competitor and Mg^2+^ (Fig. 3c) and also bound to IS*Sau2* IRR42 with a similar affinity (Fig. 3d). Crude extracts from *E. coli* with pET32a failed to bind to both left and right IS*Sau2* inverted repeat sequences as shown in lane 2 of Fig. 3c-d. Therefore, the binding was specifically carried out by the purified IS*Sau2* transposase OrfAB.

### IS*Sau2* transposase OrfAB cleaved transposon ends

**Fig. 4:**
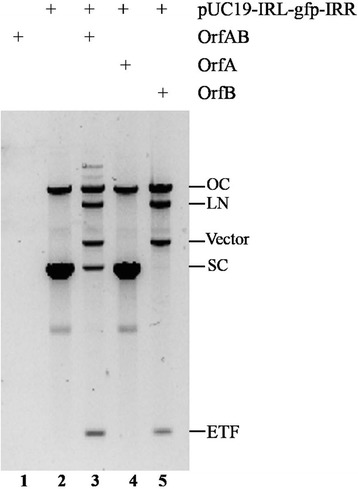
In vitro excision of IS*Sau2*. The artificial mini-transposon pUC19-IRL-gfp-IRR was incubated for 16 h in the absence or presence of OrfAB, OrfA or OrfB. Products were separated by agarose gel electrophoresis. SC, LN and OC represent supercoiled, linear and open circle pUC19-IRL-gfp-IRR, respectively; Vector stands for the vector backbone; ETF stands for the excised mini-transposon fragment

To test whether the IS*Sau2* transposase had a catalytic activity, a plasmid (pUC19-IRL-gfp-IRR), an artificial mini-transposon with IRL and IRR flanking a *gfp* gene, was incubated in a buffer with purified full-length transposase OrfAB. The reaction produced linearized plasmid and two fragments of the correct size to be excised mini-transposon (ETF) and the vector backbone (Fig. 4, lane 3). Restriction digestion confirmed that these products were produced by double-strand cleavage at IRR and IRL. As shown in lane 3 and 5 of Fig. 4, both the linearized plasmid (LN) and nicked plasmid (OC) were also produced by transposase OrfAB in the cleavage reaction. These results indicated that transposase OrfAB cleaved both single and double strands in vitro.

To test which domain in the transposase OrfAB is required for cleavage, purified OrfA and OrfB were used in the cleavage assay. OrfA did not have the catalytic activity to cleave transposon end (Fig. 4, lane 4), while OrfB and OrfAB had the similar activity for the cleavage (Fig. 4, lane 3 and lane 5). These results proved that OrfB was involved in the cleavage reaction and in IS*Sau2* transposition reactions.

### Measurement of transposition frequency of IS*Sau2* in *E. coli*

To measure the transposition frequency of IS*Sau2* in vivo, a modified GFP hop-on assay was used. A GFP hop-on assay plasmid pET28a-ISgfp was constructed. It contained a *gfp* gene which lacks transcriptional and translational signals and was located between IRL and IRR of IS*Sau2*. The *gfp* gene in the GFP-hopper was not expressed at its original location in the plasmid. In the presence of pET32a-OrfAB, it could be expected that expression of the IS*Sau2* transposase gene led to transposition of ISgfp, into an expressed gene in the bacterial genome in the correct orientation and reading frame, resulting in the fusion of the gene and *gfp* gene and expression of a green fluorescent fusion protein.

**Fig. 5:**
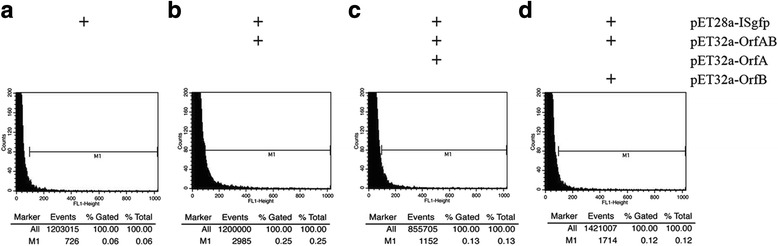
Flow cytometric analysis of ISgfp transposition and measurement of the transposition frequency of IS*Sau2*. **a** *E. coli* strain harboring pET28a-ISgfp as a negative control. **b** *E. coli* strain harboring pET28a-ISgfp and pET32a-OrfAB. **c** *E. coli* strain harboring pET28a-ISgfp, pET32a-OrfAB and pET32a-OrfA. **d** *E. coli* strain harboring pET28a-ISgfp, pET32a-OrfAB and pET32a-OrfB. Experiments were performed in triplicates and representative results from one experiment are shown. The range of gated events is indicated as marker 1 (M1)

To detect transpositional events of ISgfp by FACS, we introduced both pET28a-ISgfp and pET32a-OrfAB into *E. coli* DH5α. The strain harboring pET28a-ISgfp acted as a negative control. Per 10^6^ events through the FACS, 622.32 ± 18.84 events were detected in the negative control group (Fig. 5a). As shown in Fig. 5b, 2381.82 ± 105.68 events for 10^6^ total events were detected with the transposase induction and the number was significantly higher than that in the negative control group, demonstrating that the appearance of the extra fluorescent signals depended on the OrfAB transposase. The transposition frequency of the ISgfp, representing the transposition frequency of IS*Sau2* in vivo, was approximately 1.76 ± 0.13 × 10^− 3^.

### Effect of OrfA and OrfB on the transposition of IS*Sau2*

To investigate whether OrfA or OrfB affected the transposition activity, pET32a-OrfA and pET32a-OrfB were introduced into the strains harboring the pET28a-ISgfp and pET32a-OrfAB. As shown in Fig. 5c and D, OrfA (1209.85 ± 3.66 events for 10^6^ total events) and OrfB (1341.95 ± 4.32 events for 10^6^ total events) significantly decreased the transposition frequency of ISgfp. These data suggested that OrfA and OrfB were the inhibitors for the transposition of IS*Sau2*.

## Discussion

Until now, only 138 insertion sequences have been identified as members of IS*150* subgroup in the IS*3* family (data from ISfinder). There were only a few studies on IS*150* insertion sequences. Early studies identified an unknown insertion sequence based on the homology analyses of nucleotide sequences or protein sequences [13, 14]. Later on, presumed members have also been required to possess the common features of IS*150*: 1200–1400 bp in length, containing two *orf*, a HTH motif, a D (57–60) D (35) E motif, and an A_n_G frameshifting window (OrfAB as a transposase) [4, 9]. In accordance with these, our results confirmed that IS*Sau2* belongs to the IS*150* subgroup of IS*3* family not only supported by the sequences analysis (Fig. 1) but also by results from the molecular experiments (Fig. 3a). First, IS*Sau2* contains two open reading frames encoded two proteins. Secondly, OrfA contains a HTH motif, while OrfB contains a D (60) D (35) E motif and the OrfAB transposase produced by programmed − 1 translational frameshifting with the motif A_6_G (Fig. 1). Additionally, three proteins were expressed with similar concentrations (Fig. 3a) and this is consistent with the idea that the frequency of frameshifting in IS*150* is approximately 50% [15]. Last, the classification was further confirmed by the phylogenetic analysis using OrfB protein sequence (Fig. 2).

IS*Sau2* was first found in HA-MRSA252 strain by the complete genome sequence analysis [16]. Our previous study found that IS*Sau2* was only present in *S. aureus* [1]. Except our work, only anther paper analyzed the insertion sites of IS*Sau2* in CC30 *S. aureus* by bioinformatics analysis [8] without any other functional studies. Our results in this study confirmed that IS*Sau2* was at least partially functional with binding and cleavage activities by the transposase OrfAB and the transposase was able to catalyze the transposition in vivo.

The IS*Sau2* transposase OrfAB bound to the inverted repeat sequences, where it carries out cleavage and strand transfer reactions. The conserved HTH motif in the N terminal of OrfAB is supposed to bind the IRs specifically. The low binding of purified OrfAB to the IRL or IRR (Fig. 3c and d) was presumably due to the fact that the full-length transposase binds poorly to the ends in vitro [17]. Additionally, we showed that OrfAB was able to cleave the transposon ends, otherwise, OrfB alone could also cleave the transposon in vitro (Fig. 4) with an explicable reason that it contained the DDE domain which catalyzes transposition reactions [3]. Our results at least partly support the idea that the transposition occurred by a cut and paste mechanism like other members in the IS*3* family [18].

The transposition frequency of IS*Sau2* was estimated using a modified “GFP hop-on assay” method, which has been used to determine the transposition of transposons in both *Bacillus subtilis* and *E. coli* [19, 20]. This method, making it possible to detect transposition at the single cell level, was more efficient to estimate the transposition frequency than other methods [19, 20], such as papillation assays [21, 22], mutation accumulation experiment [23] and mating-out assay [24]. Thus, the GFP hop-on assay was adapted to measure the transposition frequency of IS*Sau2* in the presence of OrfA, OrfB or OrfAB. As shown in Fig. 5, OrfAB of IS*Sau2* induced transposition at the frequency of 1.76 ± 0.13 × 10^− 3^. Nevertheless, the observed transposition frequency (1.76 ± 0.13 × 10^− 3^) might be over-estimated than the real transposition in bacteria, due to the laboratory conditions which had few limiting factors and thus favored the transposition. In nature, the transposition rates of insertion sequences generally must be maintained at a low level, acting as a possible strategy to limit any negative effects on the host genome [25]. These data demonstrated that the transposition of IS*Sau2* depended on the transposase OrfAB and indicated that IS*Sau2* was at least partially functional.

It is interesting that both OrfA and OrfB inhibited significantly the transposition of IS*Sau2* induced by OrfAB. In the IS*3* family, OrfA inhibits reactions promoted by transposases by binding to transposon ends [26]. OrfB might form a complex with OrfA to inhibit the transposition of IS*Sau2* by blocking formation of an active transpososome consisting of transposase, two terminal IRs and target DNA or by preventing the transposase from catalyzing the strand transfer reaction [26].

In summary, we characterized a functional insertion sequence IS*Sau2* in *S. aureus*. To the best of our knowledge, this is the first study to investigate the transposition of IS*Sau2* and to characterize the effects of OrfA and OrfB of IS*Sau2* on transposition. Our results provide a solid basis for future studies of the molecular mechanisms involved in IS*Sau2* transposition and its biological function in its host *S. aureus*. Meanwhile, we now have an experimental system that allow us to characterize any novel insertion sequence in bacterial cells and even in other eukaryotic cells especially for the insertion sequences which contain two *orf* genes and use the fusion OrfAB as the transposase.

## Conclusion

We have confirmed that IS*Sau2* is a member of IS*150*/IS*3* family. The IS*Sau2* transposase OrfAB could bind to and cleave the specific fragments containing the terminal inverted repeat sequences and induce the transposition, suggesting that IS*Sau2* was functional. Meanwhile, both OrfA and OrfB were the inhibitors for transposition of IS*Sau2*. Our results will help understand biological roles of IS*Sau2* in its host *S. aureus*.

## Methods

### Construction of prokaryotic expression vectors

A known IS*Sau2* transposase sequence, located in *sdrC* of *S. aureus* E48 [27], was isolated by PCR with the specific primers pairs ISSau2-F/ISSau2-R (Table 1) using the *Pfu* polymerase, and inserted between *Bam*H I and *Xho* I sites of pET28b to create pET28-ISSau2. Within pET28-ISS*au2*, an N-terminal 6-His tag and a C-terminal 6-His tag were linked to either side of the IS*Sau2* sequence. Meanwhile, a G residue was inserted downstream of A_6_G in the IS*Sau2* sequence, forming an A_6_G_2_ region [2], by overlap PCR using the two primer pairs ISSau2-F/OrfAB-overlap-R and OrfAB-overlap-F/ISSau2-R (Table 1). In theory, addition of the G would not change the OrfAB amino acid sequence [28]. The mutated sequence was inserted between *Bam*H I and *Xho* I sites of pET32a, which contains an N-terminal 6-His tag, to create pET32-OrfAB. The *orfA* region (753 bp) and *orfB* region (891 bp) of IS*Sau2* were amplified by PCR using the primers ISSau2-F/OrfA-R and OrfB-F/ISSau2-R respectively (Table 1) and inserted into pET32a to construct pET32a-OrfA and pET32a-OrfB.

**Table 1:** Plasmids and primers used in this study^a^

Plasmid	Genotype/Description
pET28-ISSau2	pET28b derivate with IS*Sau2* transposase sequence under control of T7 promoter, Kan^R^
pET32-OrfAB	pET32a derivate with IS*Sau2* transposase sequence with an insertion of G residue in the A_6_G_1_ region to generate the A_6_G_2_ region, Amp^R^
pET32a-OrfA	pET32a derivate with OrfA sequence (the first *orf* of IS*Sau2*, 753 bp in length), Amp^R^
pET32a-OrfB	pET32a derivate with OrfB sequence (the second *orf* of IS*Sau2*, 891 bp in length), Amp^R^
pUC19-IRL-gfp-IRR	pUC19 derivate with the gfp sequence from pGLO vector flanked by IRL (42 bp) and IRR (42 bp) of IS*Sau2*, Amp^R^
pET28a-ISgfp	pET28a derivate with the gfp sequence from pGLO vector flanked by IRL (42 bp) and IRR (42 bp) of IS*Sau2*, Kan^R^
Primer	Sequence 5′-3’
ISSau2-F	CGCGGATCCATGAAAACTTTGAAGGGAGC
ISSau2-R	CCGCTCGAGCATTAAAAATGGCTGATTCTGTA
OrfAB-overlap-R	ATGCTTCAAACCTTTTTTAAAACGT
OrfAB-overlap-F	ACGTTTTAAAAAAGGTTTCAAGCAT
OrfA-R	CCGCTCGAGACGCTTCAATCGTTTTGTAT
OrfB-F	CGCGGATCCCGTTTTAAAAAAGTTTCAAGCA
IRL-gfp-F	CGCGGATCCTGAAATGCTCCCTTCAAAGTAGACATTGAAAAAATGAAAACTAT GGCTAGCAAAGGAGAA
IRR-gfp-R	CCGGAATTCAATTTTAATTATTTAATTGTCTACTAGACTGGGTGCAGTTCATTTG TAGAGCTCATCCAT
D-orfA-F	ATGAAAACTTTGAAGGGAGC
D-orfA-R	ACGCTTCAATCGTTTTGTAT
D-orfB-F	CGTTTTAAAAAAGTTTCAAGCA
D-orfB-R	CATTAAAAATGGCTGATTCTGTA
D-ISgfp-F	AATGAAAACTATGGCTAGCAA
D-ISgfp-R	TGCAGTTCATTTGTAGAGCT

^a^All the plasmids were constructed and all primers were designed in this study

### Expression and purification of IS*Sau2* transposase

The constructed expression vectors were transformed into BL21 (DE3) competent cells, separately. The overnight culture of BL21 (DE3) was diluted 1:50 in fresh LB broth containing different antibiotics (pET28-ISSau2: 50 μg/mL kanamycin; pET32-OrfAB: 100 μg/mL ampicillin) with vigorous shaking at 37 °C to an OD_600_ of 0.4–0.6. Expression of the transposase was induced by the addition of Isopropyl-β-Dthiogalactopyranoside (IPTG) to 0.2 mM. Bacterial cells were grown for 6 h at 28 °C and harvested by centrifugation.

All the following steps were carried out on ice. Cultured bacterial cells (BL21 with pET32-OrfAB) were suspended in a PBS buffer (137 mM NaCl, 2.7 mM KCl, 10 mM Na_2_HPO_4_, and 2 mM KH_2_PO_4_) and disrupted by an Ultrasonic Processor. The supernatant containing soluble transposase OrfAB was collected by centrifugation at 13000 g for 45 min. After filtration by 0.22 μm filter, the supernatant was applied to a nickel-nitrilotriacetic acid (Ni-NTa) column equilibrated with PBS. The column was washed with 10 column volumes of PBS followed by 6 column volumes of PBS plus 20 mM imidazole, impure proteins were eluted in PBS plus 75 mM imidazole and the purified OrfAB was obtained in PBS plus 500 mM imidazole. The dialyzed protein was made up to 50% glycerol and stored at − 80 °C.

### Electrophoretic mobility shift assays (EMSA)

Two 42 bp biotin-labeled DNA fragments containing IRL and IRR were generated by oligonucleotide hybridization (heated together at 95 °C for 10 min and cooled to room temperature) respectively to generate dsDNA IRL42 and IRR42. For EMSA, 2 μL 10 × binding buffer, 1 μg/μL Poly (dI-dC), 50% Glycerol, 1% NP-40, 1 M KCl, 100 mM MgCl_2_, 200 mM EDTA (EMSA kit, Thermo) and 0.42 μg OrfAB were incubated at room temperature for 20 min. Binding was initiated by addition of biotin end-labeled target DNA. Binding reactions were allowed at room temperature for 20 min and the products were then run on 5% polyacrylamide gels in 0.5 × TBE buffer (100 V) at 0 °C. The biotin end-labeled DNA was detected using the Streptavidin-Horseradish Peroxidase Conjugate and the Chemiluminescent Substrate.

### In vitro transposition assays

An in vitro transposition assay was performed as described [29]. The artificial mini transposon plasmid (pUC19-IRL-gfp-IRR) contained IRL42 and IRR42 flanking the GFP gene which lacks transcription and translation start signals, the fusion sequence was amplified by PCR using the specific primer pairs IRL-gfp-F/ IRL-gfp-R (Table 1) with the pGLO plasmid DNA as the template. The *gfp* gene was amplified so as to remove the transcriptional and translational signals. Standard in vitro transposition assays contained 0.12 pmol of pUC19-IRL-gfp-IRR in 20 mL of transposase assay buffer (10 mM MgCl_2_, 1 mM DTT, 0.1 mg/mL BSA, 50 mM Tris-HCl pH 7.5 and 50 mM NaCl). Reactions were started by the addition of 1–2 pmol of purified transposase OrfAB and incubated at 30 °C for 16 h. Reactions were stopped by heating at 75 °C for 10 min, and products were analyzed by agarose gel electrophoresis.

### Detection of GFP-hopper transposition

The plasmid pET28a-ISgfp was generated from pUC19-IRL-gfp-IRR. The GFP hop-on assay was performed as described [20] with minor modifications. The plasmid pET28a-ISgfp as well as pET32a-OrfAB or pET32a-OrfA or pET32a-OrfB was introduced into *E. coli* DH5α. The transformed colonies were cultured in 5 mL LB medium under the selective pressure (both 50 μg/mL kanamycin and 100 μg/mL ampicillin) at 37 °C. Harvested cells were then resuspended in a sheath fluid (BD Biosciences, USA) at OD_600_ = 0.3. Flow cytometry was performed on a FACSCalibur cytometer with the CELLQUEST software (BD Biosciences). GFP fluorescence was detected with a FL1 detector and its intensity was expressed as an arbitrary logarithmic value (FL1-H) within the range of 10^0^–10^4^. The transposition-frequency was estimated as the ratio between the number of events within region M1 (> 10^2^ FL1- H) in the presence of OrfAB minus the background number and the number of total events.

To investigate whether OrfA or OrfB has the activity to inhibit the transposition of ISgfp induced by OrfAB, the pET32a-OrfA or pET32a-OrfB was first introduced into *E. coli* DH5α and confirmed by PCR with the primer pairs OrfA-F/OrfA-R or OrfB-F/OrfB-R (Table 1) and then the pET28a-ISgfp and pET32a-OrfAB were introduced into *E. coli* DH5α by electroporation and confirmed by PCR using the primers ISgfp-F/ISgfp-R and OrfA-F/OrfB-R (Table 1). The GFP fluorescence of the cells containing pET28a-ISgfp, pET32a-OrfAB and pET32a-OrfA (or OrfB) were detected by FACSCalibur cytometer.

## Abbreviations

DDE: Aspartate-Aspartate-Glutamate
*E. coli*: *Escherichia coli*
EMSA: Electrophoretic Mobility Shift Assays
FACS: Fluorescence Activated Cell Sorting
GFP: Green Fluorescent Protein
HA-MRSA: Healthcare-Associated Methicillin Resistant *Staphylococcus aureus*
HTH: Helix-Turn-Helix
IPTG: Isopropyl-β-Dthiogalactopyranoside
IRs: Inverted Repeat sequences
ORF: Open Reading Frame
PBS: Phosphate Buffer solution
*S. aureus*: *Staphylococcus aureus*
